# Editorial: Using Small Molecules to Treat Macromolecule Storage Disorders

**DOI:** 10.3389/fcell.2020.623613

**Published:** 2020-11-27

**Authors:** Or Kakhlon, Pablo V. Escriba, Hasan O. Akman, Miguel Weil

**Affiliations:** ^1^Department of Neurology, Hadassah-Hebrew University Medical Center, Jerusalem, Israel; ^2^Laboratory of Molecular Cell Biomedicine, Department of Biology, University of the Balearic Islands, Palma de Mallorca, Spain; ^3^Department of Neurology, Columbia University Medical Center, New York, NY, United States; ^4^Laboratory for Neurodegenerative Diseases and Personalized Medicine, The Shmunis Schools of Biomedicine and Cancer Research, The George S. Wise Faculty for Life Sciences, Sagol School of Neurosciences, Tel Aviv University, Tel Aviv, Israel

**Keywords:** small molecules, aggregates, aggregate clearance, cell injury, storage disorders

This Research Topic focuses on small molecule therapy to various macromolecule storage disorders. Apart from nucleic acids, all macromolecular building blocks of cells—proteins, lipids, and glycans—are prone to aggregation upon chemical modification or imbalance between their synthesis and breakdown. Unmanaged, this aggregation and often ensuing precipitation can lead to cell stress and pathology. Different treatment strategies, ranging from immunotherapy to pharmacological and gene therapy, have been applied to clear these pathogenic macromolecular aggregates. In particular, the elimination of precipitated misfolded proteins by molecular chaperones has been thoroughly investigated. Lipids and glycans also precipitate into lipofuscins and polyglucosans. However, these inclusions also include oxidation-prone proteins. This Research Topic deals with pharmacological interventions aimed at countering the detrimental effects of macromolecular aggregates. Through illustrating the beneficiary or even toxic effects of different small molecules and pharmacological agents, the selected articles provide a comprehensive picture of interventional and innate means of curative aggregate clearance.

Three papers discuss amyloids, which are among the most studied protein inclusions. The Parets et al. paper introduces a novel therapeutic approach for Alzheimer's disease (AD): Instead of targeting the amyloid cascade, according to which proteolysis of the integral membrane protein amyloid precursor protein (APP) by γ-secretase is the culprit of AD (Selkoe and Hardy, 2016), the authors focus on the changes in membrane lipid composition and structure as triggers of AD pathology. They conclude that 2-hydroxy-docosahexaenoic acid (H-DHA) improves cognition in AD modeling mice by converting to the brain permeable omega-3 polyunsaturated fatty acid (PUFA) heneicosapentaenoic acid (HPA). Mechanistically, Parets et al. suggest that the neuroprotective effects associated with HPA are associated with its conversion to other omega-3 PUFAs which, in turn, could enrich brain membranes with liquid-disorder promoting lipids associated with beneficial effects against AD (Vetrivel and Thinakaran, 2010; Shaikh, 2012). Interestingly, the β-amyloid aggregate is not directly targeted in this paper, even though the authors do propose to test the effects of H-DHA on β-amyloid accumulation.

The more direct, classical, approach to amyloids as AD culprit is presented by Viswanathan et al.. This review summarizes *in vitro* and *in vivo* results showing the effects of the well-investigated small molecule Naphthoquinone Tryptophan (NQTrp) on amyloid synthesis and degradation. The approach presented is based on targeting the aggregates, rather than the cellular pathways which generate or disintegrate them. Interestingly, the review shows that through specific interaction with the misfolded form of various amyloidogenic proteins and with deposited amyloid fibrils, NQTrp can respectively inhibit the formation or disaggregate toxic amyloids.

The research paper by Lahiani-Cohen et al. is the only paper in this Topic which addresses the pathogenic, rather than the therapeutic, value of small molecules. Tau proteins regulate microtubule-dependent processes in neurons, such as neurite outgrowth and axonal transport. However, hyperphosphorylated Tau form pathological and insoluble neurofilament tangles implicated in AD, Parkinson's disease (PD) and dementia. Lahiani-Cohen et al. show that mitochondrially-generated oxidative stress induced by the small molecule 3-nitropropionic acid (3NP) can cause hyperphosphorylation and consequent neurotoxic tangle formation in both Tauopathy-modeling and wild type mice. This paper demonstrates the link between mitochondrial oxidative stress and toxic neurological aggregates and further shows that the oxidative stress-aggregopathgy axis can be facilitated by natural small molecule toxins such as 3NP.

Metabolic dysregulation can cause lipids to aggregate, chiefly in atherosclerotic plaques and sphinglolipid lysosomal deposits. Castellano et al. show that nutraceutical and dietary lipids can systemically regulate transport and deposition of lipids. Small lipid molecules such as phytosterols, carotenoids, terpenoids, and tocopherols are involved in triacylglycerol absorption and fatty acid mobilization and storage in adipocytes. Consequently these naturally occurring lipids are pivotal systemic metabolic regulators. Moreover, through controlling lipid peroxidation, these natural lipids can act as strong antioxidants and restrict adipocyte-mediated inflammation. While this review doesn't address aggregate handling, it well demonstrates lipid involvement in all aspects of metabolic regulation.

Indeed the broad metabolic implications of lipids are illustrated in Han et al.'s review. This review reveals that the lysosomal glycosphinglolipid hydrolase glucocerebrosidase also interacts with α-synuclein monomers. Therefore, glucocerebrosidase deficiency leads to aggregation of both Gaucher disease-associated glucocerbrosides and PD-associated α-synuclein oligomers. Furthermore, the latter can bind and further inhibit glucocerebrosidase in a positive feedback causing overaccumulation of α-synuclein aggregates (Lewy bodies) and glucocerbrosides (Stirnemann et al., 2017). Interestingly, because glucocerebrosidase has two aggregatogenic substrates, novel small molecule chaperones of the enzyme, such as iminosugars and ambroxol, have been developed as co-therapies for both Gaucher and PD. The Gaucher-Parkinson duality is a unique case of small molecule therapeutics which can disassemble both proteinaceous and lipidic pathogenic aggregates.

One research paper in this Topic deals with glycan overaccumulation. Mucopolysaccharidoses (MPS) are lysosomal storage disorders caused by faulty degradation of glycosaminoglycans. In their mechanistic study Pierzynowska et al. describe a new target for the MPS drug genistein. They show that genistein can inhibit proteasomal activity, yet increase proteasome levels in MPS fibroblasts. The authors show that the genistein-mediated inhibition of proteasome activity correlated with reduced ubiquitination of several proteins destined for degradation, such as partially defective lysosomal hydrolases. These results suggest that the reduced ubiquitination stabilizes these defective lysosomal enzymes, indeed sorted for lysosomal degradation by ubiquitination (Li et al., 2015), and thus increases lysosomal degradation of glycosaminoglycans and potentiates MPS therapy.

Lastly, Palhegyi et al. present a biomedical view of autophagy as a modulator of macromolecule storage disorders. This review shows that autophagy is involved in clearing protein, lipid and glycan aggregates. The review discusses innate and pharmacological regulators of autophagy, importantly stressing not only how autophagy can clear pathogenic aggregates, but also how mutant proteins causing aggregopathies can impair autophagic protection thus exacerbating pathology—for instance, inhibition of phagophore formation and autophagosome maturation by mutated huntingtin and glucocerebrosidase, respectively. The review also presents the beneficiary effect of pharmacological inducers of autophagy for treating various storage disorders.

In conclusion, this Research Topic demonstrates the importance of small molecules for the mechanistic understanding of aggregate pathogenicity. Moreover, based on examples from different storage diseases, this Topic warrants the use of small molecules as a leading therapeutic intervention for treating storage disorders.

## Author Contributions

All authors listed have made a substantial, direct and intellectual contribution to the work, and approved it for publication.

## Conflict of Interest

The authors declare that the research was conducted in the absence of any commercial or financial relationships that could be construed as a potential conflict of interest.
